# Genomic Characteristics of Emerging Intraerythrocytic *Anaplasma capra* and High Prevalence in Goats, China

**DOI:** 10.3201/eid2909.230131

**Published:** 2023-09

**Authors:** Zhe-Tao Lin, Li-Feng Du, Ming-Zhu Zhang, Xiao-Yu Han, Bai-Hui Wang, Jiao Meng, Fu-Xun Yu, Xiao-Quan Zhou, Ning Wang, Cheng Li, Xiao-Yang Wang, Jing Liu, Wan-Ying Gao, Run-Ze Ye, Luo-Yuan Xia, Yi Sun, Na Jia, Jia-Fu Jiang, Lin Zhao, Xiao-Ming Cui, Lin Zhan, Wu-Chun Cao

**Affiliations:** State Key Laboratory of Pathogen and Biosecurity, Beijing Institute of Microbiology and Epidemiology, Beijing, China (Z.-T. Lin, L.-F. Du, M.-Z. Zhang, X.-Y. Han, Y. Sun, N. Jia, J.-F. Jiang, X.-M. Cui, W.-C. Cao);; Institute of EcoHealth, School of Public Health, Shandong University, Jinan, China (L.-F. Du, M.-Z. Zhang, B.-H. Wang, N. Wang, C. Li, X.-Y. Wang, J. Liu, W.-Y. Gao, R.-Z. Ye, L.-Y. Xia, L. Zhao);; National Health Commission Key Laboratory of Pulmonary Immunological Diseases, Guizhou Provincial People’s Hospital, Guiyang, China (J. Meng, F.-X. Yu, L. Zhan);; Guizhou Provincial Blood Center, Guiyang (X.-Q. Zhou)

**Keywords:** *Anaplasma capra*, whole-genome analysis, prevalence, phylogenetic analysis, tickborne diseases, vector-borne infections, goats, bacteria, bacterial infection, zoonoses, China

## Abstract

*Anaplasma capra* is an emerging tickborne human pathogen initially recognized in China in 2015; it has been reported in ticks and in a wide range of domestic and wild animals worldwide. We describe whole-genome sequences of 2 *A. capra* strains from metagenomic sequencing of purified erythrocytes from infected goats in China. The genome of *A. capra* was the smallest among members of the genus *Anaplasma*. The genomes of the 2 *A. capra* strains contained comparable G+C content and numbers of pseudogenes with intraerythrocytic *Anaplasma* species. The 2 *A. capra* strains had 54 unique genes. The prevalence of *A. capra* was high among goats in the 2 endemic areas. Phylogenetic analyses revealed that the *A. capra* strains detected in this study were basically classified into 2 subclusters with those previously detected in Asia. Our findings clarify details of the genomic characteristics of *A. capra* and shed light on its genetic diversity.

*Anaplasma capra* is an emerging tickborne zoonotic pathogen in the genus *Anaplasma*, family Anaplasmataceae, and was initially identified in blood samples from asymptomatic goats (*Capra aegagrus hircus*) and a febrile human patient with tick-bite history in China in 2015 (*1*). The patient infected with *A. capra* had fever, headache, malaise, dizziness, myalgia, gastrointestinal symptoms, rash, lymphadenopathy, and abnormalities in cerebrospinal fluid pleocytosis and hepatic aminotransferase. Since then, *A. capra* has been detected in various domestic animals (e.g., goats, sheeps, cattle, yaks, and dogs) (*2*–*5*) and wild animals (e.g., takins, muntjacs, water deer, musk deer, onagers, serows, and brown hares) (*6*–*10*), and in a wide range of ticks (e.g., *Ixodes persulcatus*, *Haemaphysalis longicornis*, *H. qinghaiensis*, *Dermacentor abaensis*, *D. nuttalli*, *and Rhipicephalus microplus* [*1*,*11*–*14*]) across China and around the world (*2*,*7*–*10*,*15*,*16*), posing a potential threat to the health of humans and animals.

Members of the family Anaplasmataceae have complex life cycles involving vertebrate hosts and hematophagous ticks, many of which have emerged as human pathogens. The genus *Anaplasma* was proposed according to the phylogenetic analyses based on 16S rRNA and *groEL* sequences (*17*) and initially encompassed 6 species: *A. phagocytophilum*, *A. marginale*, *A. centrale*, *A. ovis*, *A. platys*, and *A. bovis*. Subsequently, 2 candidate novel species (*A. capra* and *A. odocoilei*) and other unclassified genovariants (*1*,*18*–*20*) were included in the List of Prokaryotic Names with Standing in Nomenclature (https://www.bacterio.net) pending validation. To date, 5 *Anaplasma* species have been known to infect humans: *A. phagocytophilum*, *A. capra*, *A. ovis*, *A. platys*, and *A. bovis* (*21*). Since the *A. marginale* genome sequence was reported in 2005 (*22*), a total of 24 *A. marginale* genomes (*23*), 32 *A. phagocytophilum* genomes (*24*,*25*), 1 *A. centrale* genome (*26*), 2 *A. ovis* genomes (*27*), and 1 *A. platys* genome (*28*) have been sequenced and deposited in GenBank. Although *A. capra* has been extensively detected in ticks and animal hosts worldwide, no genome of the emerging pathogen has been determined so far, which has hindered us from better understanding its genetic features and pathogenesis. Considering *A. capra* is an intraerythrocytic pathogen and abundant in blood samples of host goats (*1*,*29*), we separated erythrocytes from the blood of infected goats to enrich the bacteria and generated the entire genome of *A. capra* using metagenome assembly to promote better understanding of this emerging pathogen, to compare the characteristics of *A. capra* genomes with previously published genomes of other *Anaplasma* and related species, and to evaluate intraspecies genetic diversity of *A. capra* in different geographic locations and tick species across China.

## Materials and Methods

### Sample Collection and Preparation

We collected EDTA blood samples from 3 flocks of goats in Shandong Province and a flock of goats in Guizhou Province, China (Appendix Figure 1), during September 2021–July 2022. Meanwhile, we prepared blood smears for some goats. We collected host-seeking ticks in the same areas where the infected goats lived by dragging white flags over vegetation. An entomologist (Y.S.) identified all ticks to the species level and developmental stage. We extracted DNA from each goat blood sample or tick by using a High Pure PCR Template Preparation Kit (Roche, https://www.roche.com) according to the manufacturer’s instructions.

### PCRs and Sequencing

We conducted a nested PCR specific for the citrate synthase (*gltA*) gene of *A. capra* (Appendix Table 1) to screen all goat blood and tick samples, as previously described (*1*). We amplified all the positive samples for *gltA* by specific PCRs targeting the 16S rRNA, *msp4*, and *groEL* genes of *A. capra* (Appendix Table 1). We sequenced all amplicons to confirm the correctness of PCR results and conducted a SYBR Green–based quantitative PCR (qPCR) targeting different regions of the *gltA* gene by using a specific primer (Appendix Table 1).

### Fluorescence In Situ Hybridization

We used fluorescence in situ hybridization (FISH) to observe the *A. capra* on blood smears. We designed the probe on the basis of the 16S rRNA full-length sequence of *A. capra* (Appendix Table 2) and labeled it with Quasar 570. We resuspended the pooled FISH probes in a final concentration of 25 μmol/L in RNase-free storage buffer, which we protected from light and stored at –20°C. We performed FISH on the prepared blood smear with a commercial kit (Biosearch Technologies, https://www.biosearchtech.com), according to the manufacturer’s instructions.

### Enrichment of *A. capra* for Genomic Sequencing

We separated erythrocytes from infected goats by conducting gradient centrifugation using cell separation solution (Eppendorf, https://www.eppendorf.com) for 20 min at 200 × *g* at 4°C. Then, we added 4 times volume of precooled (4°C) erythrocyte lysis buffer (Solarbio, http://www.solarbio.net) to the isolated erythrocytes by gentle pipetting to ensure adequate mixing. After placing the lysis solution at 4°C for 10 min, we centrifuged the solution at 350 × *g* for 10 min to remove residual blood cells. After that treatment, we maximally removed the host DNA in samples. Finally, we centrifuged the supernatant at 20,000 × *g* at 4°C for 30 min. We resuspended the pooled *A. capra* for DNA extraction by using the High Pure PCR Template Preparation Kit (Roche). We then constructed a sequencing library by using the AxyPrep MAG PCR Clean Up Kit (Fisher Scientific, https://www.fishersci.com) for an MGI sequencing set (https://en.mgi-tech.com). We prepared the sequencing library according to the Whole Genome Sequencing Library Preparation Protocol (MGI). We sequenced the paired-end libraries with a read length of 2 × 150 bp on a DNBseq-T7 platform at Grandomics Gene Technology Beijing Co. Ltd (Beijing, China).

### Genome Assembly and Comparative Analyses

We mapped the clean reads to the goat (*Capra hircus*) reference genome (GenBank accession no. GCF_001704415) by using SAMtools 1.14 (*30*) to discard host-derived reads. We de novo assembled contigs from the unmapped reads by using metaSPAdes 3.15.3 (*31*). We performed contig binning by using MetaBAT 2.15 (*32*) and evaluated assembly quality by using CheckM version 1.1.3 in linage_wf mode, which searches for universal single-copy marker genes and deduces completeness and contamination on the basis of presence and absence of these genes (*33*). We generated G+C content, genome completeness, and annotation information and depicted them by using an approach described previously (*34*,*35*). We estimated average nucleotide identity (ANI) and DNA–DNA hybridization (DDH) by using fastANI 1.32 (*36*) and GGDC (https://ggdc.dsmz.de/ggdc.php).

### Phylogenetic Analyses

We deposited in GenBank the results of the phylogenetic analysis of the whole genomes of the 2 *A. capra* strains and all the genomes of *Anaplasma* species by using Orthofinder 2.5.4 (*37*), after eliminating the poorly aligned positions and divergent regions by using Gblocks 0.91b. We aligned trimmed sequence by using Muscle 5.1 (R.C. Edgar, unpub. data, https://doi.org/10.1101/2021.06.20.449169) and constructed the phylogenetic tree by using iqtree 2.2.0.3 (*38*). Furthermore, we conducted phylogenetic analyses on *A. capra gltA*, *groEL*, 16S rRNA, and *msp4* genes obtained from infected goats and ticks by using the maximum-likelihood method in MEGA11 (*39*).

### Functional Analysis of Predicted Genes

To find difference in the Kyoto Encyclopedia of Genes and Genomes (KEGG) between the 2 strains of *A. capra* and other species in the genus *Anaplasma*, were annotated orthogroup sequences by using KOfam 1.4.0 (*40*) and illustrated them using a Venn diagram. We used the software eggNOG-Mapper 2.1.7 to determine the Clusters of Orthologous Group (COG) categories for protein encoding regions (*41*).

## Results

**Figure 1:**
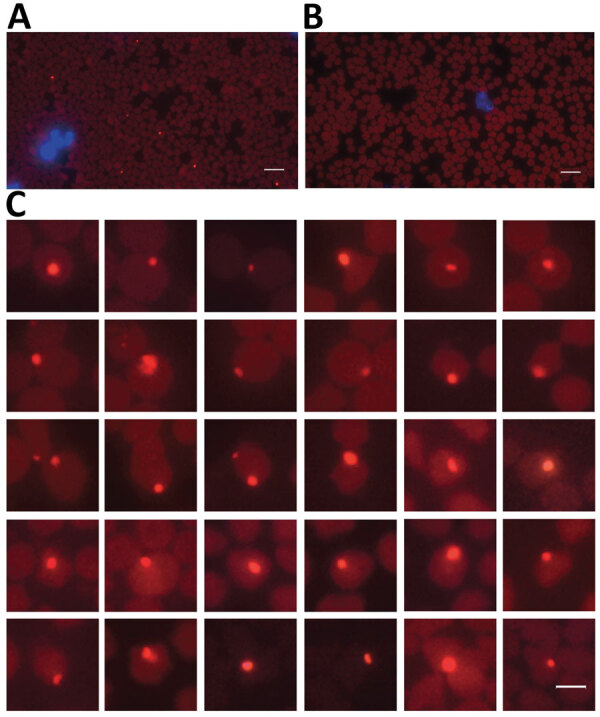
*Anaplasma capra* in the erythrocytes of an infected goat detected by fluorescence in situ hybridization (FISH) in study of emerging intraerythrocytic *A. capra* and high prevalence in goats, China. Glowing red indicates *A. capra*; blue indicates leukocyte nucleus stained with fluorescent antibody blocker containing DAPI. A) FISH results under fluorescence microscope of *A. capra*. B) FISH results of *A. capra*–negative blood smear. C) FISH results showing different shapes and sizes of *A. capra* in erythrocytes.

Forty-three (59.7%) of 72 goat blood samples were positive for *gltA* gene of *A. capra*. We chose 2 blood samples (1 from a 2-year-old female goat in Shandong Province and another from a 10-month-old female goat in Guizhou Province) (Appendix Figure 1) for next-generation sequencing because they had high bacterial loads (8.4 × 10^6^ *gltA* gene copies/mL blood for the goat in Shandong Province and 2.0 × 10^6^ *gltA* gene copies/mL blood for the goat in Guizhou Province) as estimated by qPCR (Appendix Table 1). In addition, we visualized *A. capra* by specific FISH in erythrocytes on the blood smear prepared from the goat in Shandong Province for next-generation sequencing (Figure 1).

The metagenome sequencing resulted in >38 million 150-bp clean reads from each sample. Despite primary removing of host DNA, 95.9% and 93.3% of reads in the 2 samples were mapped to the goat genome and discarded. The remaining reads were subsequently de novo assembled into contigs by using the SPAdes 3.15.3 with meta parameters (*31*). The 2 assembled *A. capra* genomes were named *A. capra* str. BIME1 (GenBank accession no. GCA_025628785.1) and *A. capra* str. BIME2 (GenBank accession no. GCA_025628805.1), and had a higher level of completeness (99.79% for BIME1 and 99.36% for BIME2). The genome of *A. capra* was the smallest (≈1.07 Mb) among those in the genus *Anaplasma* and the second smallest genome of the family Anaplasmataceae, just after *Neorickettsia sennetsu* (0.859 Mb) (*24*). The genome sequences of the 2 strains shared 99.89% nucleotide similarity with each other.

**Figure 2:**
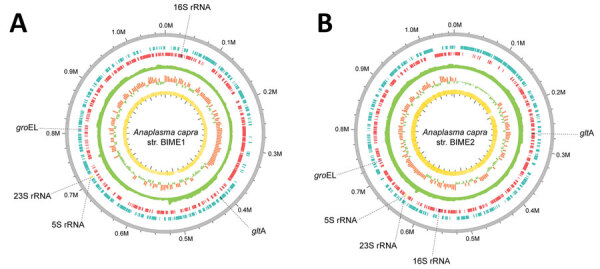
Circular map of *Anaplasma capra* strains BIME1 and BIME2 genomes in study of emerging intraerythrocytic *A. capra* and high prevalence in goats, China. The outermost ring shows the genome size in 100-kb increments. Moving inward, the blue-green and red marks indicate the coding sequences on the reverse and forward strands. The fourth ring represents the sequencing depth. The fifth ring shows the G+C skew, and the sixth rings show and G+C content. The location of *groEL and* *gltA* genes and the complete ribosomal RNA genes (5S rRNA, 16S rRNA, and 23S rRNA) within the genome are indicated.

We compared the 2 *A. capra* genomes with other representative species strains in the genus *Anaplasma* (Appendix Table 3). The G+C content (48.3% for both) of the 2 *A. capra* genomes was similar to those of *A. ovis*, *A. marginale*, *and A. centrale*, which are all intraerythrocytic pathogens. The *A. capra* genomes yielded a total of 929 and 932 genes, of which 862 and 863, respectively, represented coding sequences. They possessed 37 tRNAs and a complete ribosomal RNA operon, in which the 16S rRNA gene was separated from the 23S-5S rRNA gene pair (Figure 2) as displayed by other members of the order Rickettsiales (*42*). The 2 strains of *A. capra* and other intraerythrocytic *Anaplasma* species, including *A. ovis*, *A. centrale*, and *A. marginale*, contained comparable numbers of pseudogenes that have lost functions owing to mutation accumulation and are observed more frequently in obligate intracellular bacteria where the lost gene functions are compensated by the host cells (*43*). Of note, *A. phagocytophilum* has ≈4-fold more pseudogenes than the other *Anaplasma* species (Appendix Table 3).

**Figure 3:**
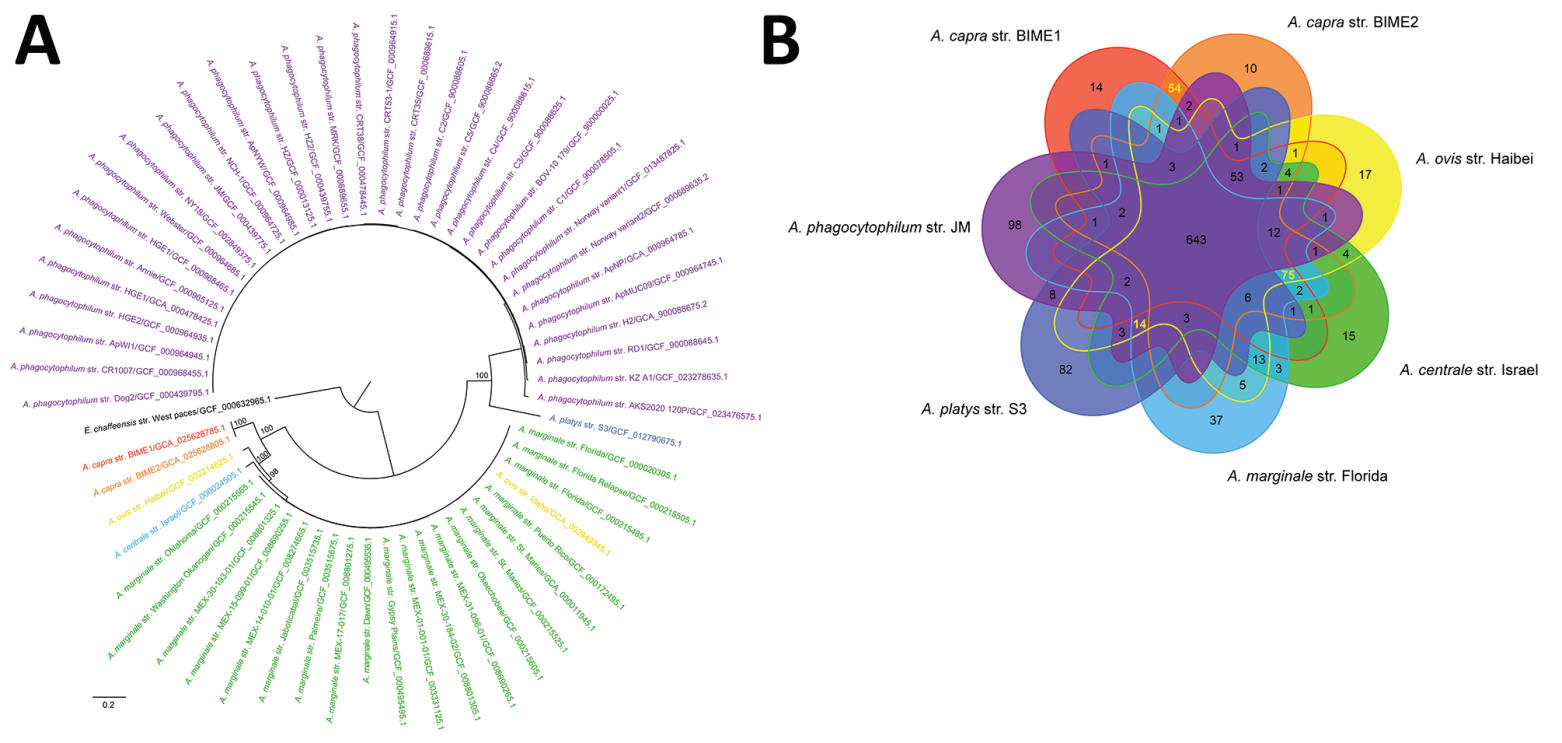
Phylogenetic tree and genomic comparison among *Anaplasma* species in study of emerging intraerythrocytic *A. capra* and high prevalence in goats, China. A) Phylogenetic tree of *Anaplasma* species based on all the genomic sequences deposited in GenBank, constructed by using maximum-likelihood method with *Ehrlichia chaffeensis* as an outgroup. The percentages of replicate trees in which the associated taxa clustered together in the bootstrap test (1,000 replicates) are shown next to the branches. B) Differences in gene contents among *Anaplasma* species strains. Venn diagrams show the distribution of shared and unique gene clusters among representative *Anaplasma* species.

The estimated values of ANI and DDH between *A. capra* and other *Anaplasma* species suggested that *A. capra* were distinct from the other species. On the basis of ANI values, *A. capra* str. BIME1 was most similar to *A. marginale*, whereas *A. capra* str. BIME2 was most similar to *A. ovis*. The DDH results revealed that both *A. capra* strains were most close to *A. marginale* (Appendix Table 4). The phylogenetic analysis based on the single copy genes revealed that the 2 *A. capra* strains together occupied a distinct branch and were more closely related to *A. ovis*, *A. marginale*, and *A. centrale* than to *A. phagocytophilum* and *A. platys* in the genus *Anaplasma* (Figure 3, panel A). To explore the gene differences in species in the genus *Anaplasma*, we used Orthofinder (*37*) to identify the homologous genes. All species in the genus *Anaplasma* shared 643 genes in common, and the 2 *A. capra* strains together with other intraerythrocytic *Anaplasma* species (*A. ovis*, *A. centrale*, and *A. marginale*) shared 75 genes that are not present in the other 2 species, *A. phagocytophilum* and *A. platys*. Compared with other members of the genus *Anaplasma*, 14 genes were not possessed by *A. capra*. Of note, a total of 54 genes were only shared by the 2 *A. capra* strains, which had other 14 distinct genes in BIME1 and 10 in BIME2 (Figure 3, panel B). In addition, we identified 25 virulent genes in the 2 *A. capra* strains that were shared by all the species in the genus of *Anaplasma*, including *virB2* gene family, *virB6* gene family, *virB4* gene family, *virB8* gene family, *virB9* gene family, and *virB3*, *virB7*, *virB10*, *virB11*, *virD4*, *and Ats-1* genes that encode the type 4 secretion system and membrane protein-encoding genes (Appendix Table 5).

Among the 54 unique genes of *A. capra*, a total of 37 were unclassified, none of which was assigned to any KEGG category. Six of the remaining 17 genes were associated with metabolic processing, 5 genes were related to genetic information processing, and 6 were involved in signaling and cellular processing (Appendix Table 6). Among them, the most noteworthy of genes were *RSF1*, a gene related to the repair of DNA double-strand breaks (*44*), and *desk*, which encodes a protein acting as a kinase at cold temperatures in *Bacillus subtilis* (*45*).

We classified the coding proteins of the 2 *A. capra* strains (BIME1 and BIME2) into functional clusters of orthologous group (COG) categories and compared them with those of representative species strains in the genus *Anaplasma* (Appendix Table 7). Most proteins were involved in translation, ribosomal structure and biogenesis, energy production and conversion, and nutrient (including amino acid, nucleotide, carbohydrate, coenzyme, and lipid) transport and metabolism, all of which were essential for bacterial survival. Of note, the number of genes encoding cell wall and membrane in *A. platys* was substantially lower than those of other *Anaplasma* species. In addition, ≈10% of the proteins did not assign to any COG category and were classified as function unknown in each species.

We screened blood samples from 3 flocks of 54 goats in Shandong Province and a flock of 18 goats in Guizhou Province (Appendix Figure 1) by using nested PCR and qPCR targeting different regions of the *gltA* gene (Appendix Table 1). The overall positive rate was 59.7% (95% CI 48.4%–71.0%), and the positive rate was significantly higher among goats in Guizhou Province than in Shandong Province (77.8% vs. 53.7%; p<0.001). Accordingly, among the *H. longicornis* ticks collected from the same sites of the positive goats, the overall positive rate was 8.0% (95% CI 4.2%–11.8%), and the *A. capra* infection rate was significantly higher among ticks in Guizhou Province than that in Shandong Province (15.8% vs. 4.9%; p<0.001) (Appendix Table 8). To understand the genetic diversity, we amplified *A. capra* 16S rRNA (1,500 bp), *groEL* (1264 bp), and *msp4* (799 bp) genes from those positive samples. We compared the nucleotide identities for each gene sequence and (Appendix Figures 2–5; GenBank accession numbers are provided).

**Figure 4:**
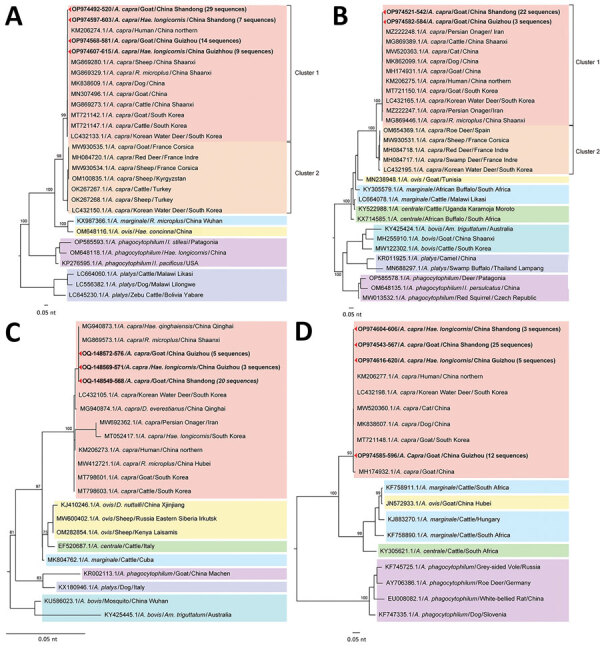
Phylogenetic analysis of *Anaplasma capra* based on nucleotide sequences of 4 genes in study of emerging intraerythrocytic *A. capra* and high prevalence in goats, China. A) Phylogenetic tree based on 536 bp nucleotide sequence of *gltA*. B) Phylogenetic tree based on 620 bp nucleotide sequence of *groEL*. C) Phylogenetic tree based on 860 bp nucleotide sequence of 16S rRNA. D) Phylogenetic tree based on 642 bp nucleotide sequence of *msp4*. We performed bootstrap analysis of 1,000 replicates to assess the reliability of the reconstructed phylogenies. GenBank accession numbers are provided. Scale bars show estimated evolutionary distance.

The *gltA* genes amplified from either goats or ticks in this study had 99.7%–100% identity with each other and with the strain that infected humans (Appendix Figure 2). The phylogenetic analysis based on *gltA* gene revealed that the *A. capra* sequences in this study were in an independent cluster from those previously reported in various animals from China and South Korea but distinct from those detected in wild and domestic animals from Europe and Kyrgystan. The South Korea water deer seemed to be capable of carrying both variants of *A. capra* (Figure 4, panel A). No *A. capra groEL* gene was acquired from tick samples, and the sequences from goats shared 99.4%–100% identity with each other and 99.8%–100% with sequences from humans (Appendix Figure 3). Similarly, the phylogenetic analyses based on the *groEL* gene revealed that *A. capra* strains of this study clustered with those from humans, dogs, and domestic ruminants in Asia but were distinguished from those in Europe (Figure 4, panel B). The entire 16S rRNA gene sequences (1,500 bp) of *A. capra* detected in goats and *H. longicornis* ticks from either Shandong or Guizhou Province shared average similarity of >99.7% from each other and from the sequence detected in humans (Appendix Figure 4). The phylogenetic tree based on 16S rRNA gene sequences indicated that all the *A. capra* strains detected in this study were in the same clade with previously reported strains in Asia (Figure 4, panel C). The *A. capra msp4* gene sequences were also relatively conserved (Appendix Figure 5) among the goats and ticks, and the topology of phylogenetic tree based on *msp4* gene were similar to that based on the 16S rRNA gene, in which all *A. capra* sequences clustered in the clade different from other members of *Anaplasma* species (Figure 4, panel D).

## Discussion

Whole-genome assembly of obligate intracellular bacteria has usually been hindered by the DNA presence of host cells. In this study, we first assembled 2 complete genomes of *A. capra* from the red blood cells of infected goats by using the metagenomic sequencing strategy. Because *A. capra* is an intraerythrocytic pathogen (*1*,*29*), we separated erythrocytes from the periphery blood of the infected goats and then lysed them for maximum removal of goat DNA. After metagenomic next-generation sequencing, we discarded the remaining goat genomic sequences and successfully assembled the *A. capra* genomes from 2 infected goats. The high percentage of reads from goat could be attributable to the low abundance of *A. capra* in erythrocytes or the fact that all other host cells rather than erythrocytes were not totally removed during the isolation of erythrocytes. In any case, the completeness of the 2 *A. capra* genomes are up to 99.79% for BIME1 and 99.36% for BIME2. The genome sizes obtained in this study reach 1,066,874 bp for BIME1 and 1,059,758 bp for BIME2. Therefore, their predicted sizes are ≈1.07 Mbp, which remain the smallest genome in the genus of *Anaplasma*. The phylogenetic analysis based on genome sequences and the comparative analyses of genomic characteristics provide the evidence that *A. capra* is closely related to other intraerythrocytic *Anaplasma* species, including *A. ovis*, *A. centrale*, and *A. marginale*.

The genome of *A. capra* consists of a single circular chromosome with a total size of 1.07 Mbp and has 862 protein-coding genes, which is smaller than other *Anaplasma* species. In fact, all the *Anaplasma* genomes sequenced so far are relatively small compared with free-living bacteria. The small genome size might be because a part of the intracellular bacterial functions has been compensated by the host cells, a process of reductive evolution that has occurred in the order Rickettsiales because of long-term intracellular association with eukaryotic hosts (*46*). This reductive evolution is associated with the frequent formation of pseudogenes, affecting distinct loci in different species (*47*). Moreover, we found that the G+C content of *A. capra* is close to that of *A. ovis*, *A. marginale*, and *A. centrale*. Of note, their relatedness also seems to be closest according to the phylogenetic analysis. The common invasiveness of erythrocytes also accounts for their high similarity.

A limitation of this study is that both the *A. capra* genomes were directly derived from the blood samples of infected goats through metagenomic next-generation sequencing. Unfortunately, we did not obtain the genomes at chromosome level, which usually relies on 3rd-generation sequencing of an isolate. In any case, this study reveals the genomic characteristics of *A. capra* and sheds light on its genetic diversity.

The high prevalence of *A. capra* in goats from Shandong and Guizhou Provinces in this study further indicate that domestic ruminants might be the main animal hosts, as suggested by previous studies (*2*–*5*). *H. longicornis* ticks collected from the same sites of the positive goats either in Shandong Province or Guizhou Province are naturally infected with *A. capra*, implying the role of the tick species in transmission of the pathogen. Phylogenetic analyses based on the *gltA* and *groEL* genes demonstrate that *A. capra* strains detected from goats and *H. longicornis* ticks in this study are clustered in the same clade with those from humans, domestic ruminants, dogs, and Korean water deer (*2*,*3*,*5*,*10*). Of note, another clade of *A. capra* strains is mainly found in the wild and domestic animals from Europe and Kyrgyzstan (*6*,*10*,*48*). Those findings suggest that the enzootic cycles in various regions of the world might be different. Public health professionals should pay enough attention and formulate prevention and control strategies to reduce the health threat of the emerging tickborne pathogen to humans in other countries besides China.

Appendix Additional information about genomic characteristics of emerging intraerythrocytic *Anaplasma capra* and high prevalence in goats, China.
