# Supplementary material for: Genomic Characteristics of Emerging Intraerythrocytic Anaplasma capra and High Prevalence in Goats, China
Source: Emerg Infect Dis. 2023 Sep;29(9):1780–8. doi: 10.3201/eid2909.230131 (PMC10461651; doi:10.3201/eid2909.230131)
Supplement: Appendix — Additional information about genomic characteristics of emerging intraerythrocytic Anaplasma capra and high prevalence in goats, China. [file 23-0131-Techapp-s1.pdf]

Article DOI: https://doi.org/10.3201/eid2909.230131

Genomic Characteristics of Emerging Intraerythrocytic *Anaplasma capra* and High Prevalence in Goats, China

Appendix

Appendix Table 1. Nucleotide sequence of primers used in the study

Target	Primer name	Primer sequence (5′-3′)	Annealing temperature (°C)	Amplicon size (bp)	References
rrs	Eh-out1	TTGAGAGTTTGATCCTGGCTCAGAACG	50	1500	(*1*)
	3–17U	WAAGGWGGTAATCCAGC			
gltA	Outer-f	GCGATTTTAGAGTGYGGAGATTG	55	1076	(*2*)
	Outer-r	TACAATACCGGAGTAAAAGTCAA			
	Inner-f	GGGTTCMTGTCYACTGCTGCGTG	51	792	(*2*)
	Inner-r	TTGGATCGTARTTCTTGTAGACC			
groEL	Forward	GCGAGGCGTTAGACAAGTCCATT	56	1264	(*2*)
	Reverse	TCCAGAGATGCAAGCGTGTATAG			
msp4	Forward	CAGTCTGCGCCTGCTCCCTAC	55	799	(*2*)
	Reverse	AGGAATCTTGCTCCAAGGTTA			
msp2	Forward	GCGTGTTGATGGCTCTGGT	51	1139	(*2*)
	Reverse	ACCAGTATCCTTATTTTTACC			
*gltA**	Forward	CGAATCTATTTGCCTGCTT	60	200	This study
	Reverse	ATCGTAATTCTTGTAGACCCT			

*This pair of primers is used for the quantitative PCR.

References

1. Wen B, Jian R, Zhang Y, Chen R. Simultaneous detection of *Anaplasma marginale* and a new *Ehrlichia* species closely related to *Ehrlichia chaffeensis* by sequence analyses of 16S ribosomal DNA in *Boophilus microplus* ticks from Tibet. J Clin Microbiol. 2002;40:3286–90. PubMed https://doi.org/10.1128/JCM.40.9.3286-3290.2002

2. Li H, Zheng YC, Ma L, Jia N, Jiang BG, Jiang RR, et al. Human infection with a novel tick-borne Anaplasma species in China: a surveillance study. Lancet Infect Dis. 2015;15:663–70. PubMed https://doi.org/10.1016/S1473-3099(15)70051-4

Appendix Table 2. Probe sequences of fluorescence in situ hybridization (FISH)

Probe sequence (5′ to 3′)	Probe sequence name
TTCTGAGCCAGGATCAAACT	AC16S-1
TCGACTTGCATGTGTTAAGC	AC16S-2
AGCAAGCTACAGATTTGGTC	AC16S-3
CGTCTGCCACTAACCAAATC	AC16S-4
AGATTCCTATGCATTACTCA	AC16S-5
TGGCTATCCCATACTACTAG	AC16S-6
GGATTATACGGTATTACCCA	AC16S-7
ATAGCGATAAATCTTTCCCC	AC16S-8
CCAACTAGCTAATCCGACAT	AC16S-9
ACAGATCACTGCCTTGGTAG	AC16S-10
TGATCATCCTCTCAGACCAG	AC16S-11
CATTGTCCAATATTCCCCAC	AC16S-12
CATAGCTGGATCAGGCTTGC	AC16S-13
TTTTACAACCCTAAGGCCTT	AC16S-14
TCATTATCTTCCCTACTGAA	AC16S-15
GGGACTTCTTCTGTAGGTAC	AC16S-16
CGCCCAATAATTCCGAACAA	AC16S-17
TTAACTTACCAAACCGCCTA	AC16S-18
GTTAAGCCCTGGTATTTCAC	AC16S-19
TGCAGTATTAAAAGCAGCCC	AC16S-20
TATCCTCTTCCGGACTCTAG	AC16S-21
ATTTCACCTCTACACTAGGA	AC16S-22
GTGTTCCTCCTAATATCTAC	AC16S-23
CAGGGTATCTAATCCTGTTT	AC16S-24
TCAGCACTCATCGTTTACAG	AC16S-25
CAACACAGAGGCAAAAGCCC	AC16S-26
CGGAGTGCTTAACGCGTTAG	AC16S-27
CCTTTGAGTTTTAGTCTTGC	AC16S-28
CGAATTAAACCACATGCTCC	AC16S-29
TGGTAAGGTTTTTCGCGTTG	AC16S-30
ATCTAACCTCCATGTCAAGA	AC16S-31
AACTGCGCCTTTCTGTTAAG	AC16S-32
ACGAGCTGACGACAGCCATG	AC16S-33
ACTTAACCCAACATCTCACG	AC16S-34
ATGAGGGTTACGCTCGTTGC	AC16S-35
CATTACCCGCTGGTAACTAA	AC16S-36
CACCGGCAGTTTCCTTAAAG	AC16S-37
CGTGCTGACTTGACATCATC	AC16S-38
CATTGTAGCACGTGTGTAGC	AC16S-39
CGACGTTGCAACCTATTGTG	AC16S-40
CTTTTACGGATTAGCTCAGC	AC16S-41
CTCGAGTTGCAGAGGACAAT	AC16S-42
TCCACGATTACTAGCGATTC	AC16S-43
CGAGAACGTATTCACCGTGG	AC16S-44
TGACGGGCAGTGTGTACAAG	AC16S-45
TTTGAGTTAAGCCAATTCCC	AC16S-46
CACCGACCCAACCTTAAATG	AC16S-47
TACAGCTACCTTGTTACGAC	AC16S-48

Appendix Table 3. Genomic characteristics of *Anaplasma capra* strains BIME1 and BIME2 compared with that of representative *Anaplasma* species strains*

	Strain (GenBank accession no.)						
Characteristic	*A. capra* str. BIME1 (GCA_025628785.1)	*A. capra* str. BIME2 (GCA_025628805.1)	*A. ovis* str. Haibei (NZ_CP015994.1)	*A. centrale* str. Israel (NC_013532.1)	*A. marginale* str. Florida (NC_012026.1)	*A. platys* str. S3 (NZ_CP046391.1)	*A. phagocytophilum* str. JM (NC_021880)
Size (bp)	1,066,874	1,059,758	1,214,674	1,206,806	1,202,435	1,196,811	1,481,598
GC rate (%)	48.32	48.32	48.9	50.0	49.8	45.5	41.6
Gene counts (n)	929	932	1021	993	992	940	1155
CDS counts (n)	862	863	945	922	913	882	997
Pseudogenes (n)	27	29	32	27	35	17	114
rRNAs (n)	3	3	3	3	3	3	3
tRNAs (n)	37	37	37	37	37	34	37
Completeness (%)	99.79	99.36	NA	NA	NA	NA	NA

*bp, base pair; CDS, coding sequence; rRNA, ribosomal ribonucleic acid; tRNA, transfer ribonucleic acid; NA, not applicable.

Appendix Table 4. The estimated values of average nucleotide identity (ANI) and DNA-DNA hybridization (DDH) between *Anaplasma capra* and the other *Anaplasma* species

	ANI		DDH	
Species	*A. capra* str. BIME1	*A. capra* str. BIME2	*A. capra* str. BIME1	*A. capra* str. BIME2
A. ovis	78.0783 (GCA_002849345.1)	78.0878 (GCA_002214625.1)	17.4 (GCA_002849345.1)	17.5 (GCA_002849345.1)
A. marginale	78.2897 (GCA_008801305.1)	77.9471 (GCA_000020305.1)	17.9 (GCA_000172475.1)	17.9 (GCA_000172475.1)
A. centrale	77.9688 (GCA_000024505.1)	77.8613 (GCA_000024505.1)	17.4 (GCA_000024505.1)	17.4 (GCA_000024505.1)
A. phagocytophilum	(-)	(-)	13.0 (GCA_023476575.1)	13.0 (GCA_023278635.1)
A. platys	(-)	(-)	13.1 (GCA_012790675.1)	13.1 (GCA_012790675.1)

Appendix Table 5. Virulence genes in *Anaplasma capra* str. BIME1 and BIME2

		GenBank accession number	
Gene	Description	*A. capra* str. BIME1	*A. capra* str. BIME2
virB2	type IV secretion system protein VirB2 family	MCU7611221.1<br>MCU7611222.1<br>MCU7611775.1<br>MCU7611780.1<br>MCU7611781.1<br>MCU7611782.1	MCU7612774.1<br>MCU7612775.1<br>MCU7612776.1
virB3	type IV secretion system protein VirB3	MCU7611541.1	MCU7612020.1
virB4	type IV secretion system protein VirB4 family	MCU7611542.1<br>MCU7611779.1	MCU7612019.1<br>MCU7612773.1
virB6	type IV secretion system protein VirB6 family	MCU7611543.1<br>MCU7611544.1<br>MCU7611545.1<br>MCU7611546.1	MCU7612018.1<br>MCU7612017.1<br>MCU7612016.1<br>MCU7612015.1
virB7	type IV secretion system protein VirB7	MCU7611364.1	MCU7612438.1
virB8	type IV secretion system protein VirB8 family	MCU7611203.1<br>MCU7611581.1	MCU7612293.1<br>MCU7611980.1
virB9	type IV secretion system protein VirB9 family	MCU7611202.1<br>MCU7611762.1	MCU7612294.1<br>MCU7612488.1
virB10	type IV secretion system protein VirB10	MCU7611201.1	MCU7612295.1
virB11	type IV secretion system ATPase VirB11	MCU7611200.1	MCU7612296.1
virD4	type IV secretion system component VirD4	MCU7611199.1	MCU7612297.1
Ats-1	*Anaplasma* T4SS translocated substrate-1	MCU7611426.1	MCU7612135.1
ompA	outer membrane protein OmpA	MCU7611514.1	MCU7612047.1
Asp14	14-kDa *Anaplasma* surface protein Asp14	MCU7611843.1	MCU7612563.1

Appendix Table 6. Genes predicted to be unique in *Anaplasma capra* str. BIME1 and BIME2

Gene	Protein	Function	
menA	1,4-dihydroxy-2-naphthoate polyprenyltransferase	Metabolic processing	Menaquinone (vitamin K2) biosynthesis
unknown	Glycosyltransferase 2 family protein		Glycan metabolism
MKK9	Mitogen-activated protein kinase kinase 9		Ethylene and camalexin biosynthesis
MqnX	Aminodeoxyfutalosine deaminase		Menaquinone Biosynthesis
CPS1	Peregrinol diphosphate synthase		Metabolism
atuF	Geranyl-CoA carboxylase α subunit		Geraniol degradation
Zbtb46	Zinc finger and BTB domain-containing protein 46	Genetic information processing	Transcription factor
UFL1	E3 UFM1-protein ligase 1		Cellular regulation
Hmbox1	Homeobox-containing protein 1		Transcription factor
RSF1	Remodeling and spacing factor 1		DNA repair
Ara54	E3 ubiquitin-protein ligase		Cellular regulation

Gene	Protein	Function	
MACC1	Metastasis-associated in colon cancer protein 1	Signaling and cellular processing	Signaling regulator
dia	Diaphanous protein		Cytokinesis
desK	Membrane-associated kinase DesK		Membrane-associated kinase
hbhA	Heparin binding hemagglutinin		Virulence factor
Mrgprg	Mas-related G protein-coupled receptor member G		G protein-coupled receptor
unknown	Membrane protein		Protein with domain of unknown function
unknown*	unclassified protein	Function unknown	

*Including 37 unclassified genes.

Appendix Table 7. Functional Clusters of Orthologous Groups of protein-coding genes from the representative *Anaplasma* species strains

Category	Functional category	*A. capra* str. BIME1	*A. capra* str. BIME2	*A. ovis* str. Haibei	*A. centrale* str. Israel	*A. marginale* str. Florida	*A. platys* str. S3	*A. phagocytophilum* str. JM
		Number of genes						
A	RNA processing and modification	1	1	1	1	1	1	1
B	Chromatin structure and dynamics	0	0	0	0	0	0	0
C	Energy production and conversion	67	67	74	70	68	71	74
D	Cell cycle control, cell division, chromosome partitioning	16	15	18	17	18	11	14
E	Amino acid transport and metabolism	31	31	35	34	35	30	25
F	Nucleotide transport and metabolism	54	54	55	54	54	52	56
G	Carbohydrate transport and metabolism	21	20	26	26	25	27	22
H	Coenzyme transport and metabolism	65	65	67	64	66	58	68
I	Lipid transport and metabolism	28	28	30	29	28	29	29
J	Translation, ribosomal structure and biogenesis	126	127	131	130	130	128	137
K	Transcription	18	17	21	20	21	19	21
L	Replication, recombination and repair	52	53	53	54	52	51	57
M	Cell wall/membrane/ envelope biogenesis	48	47	62	58	60	35	62
N	Cell motility	2	2	2	2	2	2	2
O	Posttranslation al modification, protein turnover, chaperones	44	44	43	43	43	43	45

Category	Functional category	*A. capra* str. BIME1	*A. capra* str. BIME2	*A. ovis* str. Haibei	*A. centrale* str. Israel	*A. marginale* str. Florida	*A. platys* str. S3	*A. phagocytophilum* str. JM
		Number of genes						
P	Inorganic ion transport and metabolism	31	31	36	35	34	35	34
Q	Secondary metabolites biosynthesis, transport and catabolism	12	12	12	12	12	11	12
R	General function prediction only	0	0	0	0	0	0	0
S	Function unknown	90	91	89	91	92	83	88
T	Signal transduction mechanisms	9	9	10	10	10	10	9
U	Intracellular trafficking, secretion, and vesicular transport	38	38	38	38	38	40	40
V	Defense mechanisms	4	4	4	4	4	4	4
W	Extracellular structures	0	0	0	0	0	0	0
X	Mobilome: prophages, transposons	0	0	0	0	0	0	0
Y	Nuclear structure	0	0	0	0	0	0	0
Z	Cytoskeleton	0	0	0	0	0	0	0

Appendix Table 8. The *Anaplasma capra*-positive numbers of goats and *Hae. Longicornis* in this study by PCR toward different gene loci

Gene loci	Goats from Shandong	Goats from Guizhou	*Hae. longicornis* from Shandong	*Hae. longicornis* from Guizhou
No. of tested	54	18	144	57
16S rRNA、*gltA*、*groEL*、*msp4*(+)	14	1	0	0
16S rRNA、*gltA*、*msp4*(+)	4	4	0	3
16S rRNA、*gltA*、*groEL*(+)	1	0	0	0
gltA、*groEL*、*msp4*(+)	7	2	0	0
16S rRNA、*gltA*(+)	1	0	0	0
*glt*A、*msp*4(+)	0	5	3	2
gltA(+)	2	2	4	4

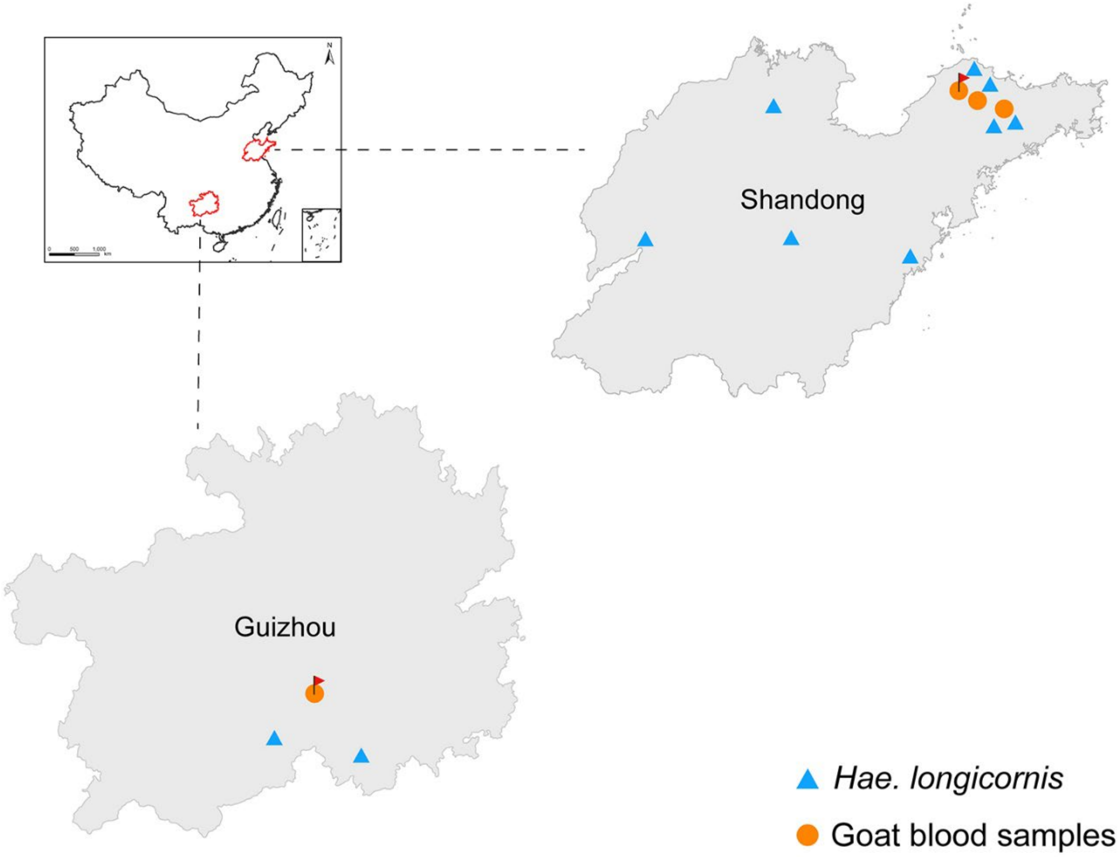

Appendix Figure 1. The sampling sites where ticks and blood samples were collected in this study. Different color and marks represent the types of samples collected in different areas. The flags indicate the locations, where the goat blood samples were collected for next-generation sequencing of *Anaplasma capra* genomes.

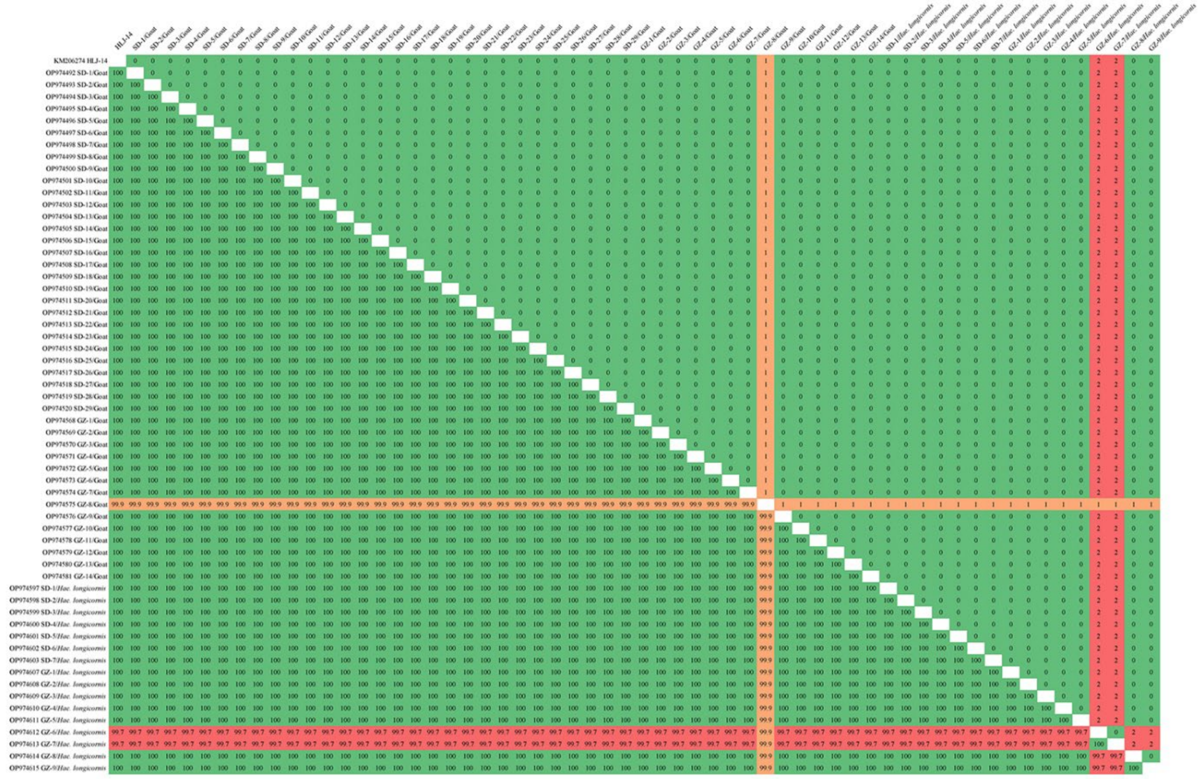

Appendix Figure 2. The comparison between each *gltA* gene sequences of *Anaplasma capra* this study and sequence from human. The upper right part represents the number of bases that differ from each sequence and the lower left part represents the nucleotide identity (%) between each sequence from others.

	HLJ-14	SD-1/Goat	SD-3/Goat	SD-4/Goat	SD-5/Goat	SD-6/Goat	SD-7/Goat	SD-9/Goat	SD-12/Goat	SD-13/Goat	SD-14/Goat	SD-15/Goat	SD-16/Goat	SD-17/Goat	SD-18/Goat	SD-20/Goat	SD-21/Goat	SD-22/Goat	SD-23/Goat	SD-25/Goat	SD-26/Goat	SD-27/Goat	SD-29/Goat	GZ-3/Goat	GZ-5/Goat	GZ-11/Goat
KM206275 HLJ-14		0	0	0	0	0	0	0	0	0	0	0	0	0	0	0	0	0	0	0	0	0	0	2	0	0
OP974521 SD-1/Goat	100		0	0	0	5	0	0	0	0	0	0	0	0	0	0	0	0	0	0	0	0	0	2	0	0
OP974522 SD-3/Goat	100	100		0	0	5	0	0	0	0	0	0	0	0	0	0	0	0	0	0	0	0	0	2	0	0
OP974523 SD-4/Goat	100	100	100		0	5	0	0	0	0	0	0	0	0	0	0	0	0	0	0	0	0	0	2	0	0
OP974524 SD-5/Goat	100	100	100	100		5	0	0	0	0	0	0	0	0	0	0	0	0	0	0	0	0	0	2	0	0
OP974525 SD-6/Goat	100	99.6	99.6	99.6	99.6		5	5	5	5	5	5	5	5	5	5	5	5	5	5	5	5	5	7	5	5
OP974526 SD-7/Goat	100	100	100	100	100	99.6		0	0	0	0	0	0	0	0	0	0	0	0	0	0	0	0	2	0	0
OP974527 SD-9/Goat	100	100	100	100	100	99.6	100		0	0	0	0	0	0	0	0	0	0	0	0	0	0	0	2	0	0
OP974528 SD-12/Goat	100	100	100	100	100	99.6	100	100		0	0	0	0	0	0	0	0	0	0	0	0	0	0	2	0	0
OP974529 SD-13/Goat	100	100	100	100	100	99.6	100	100	100		0	0	0	0	0	0	0	0	0	0	0	0	0	2	0	0
OP974530 SD-14/Goat	100	100	100	100	100	99.6	100	100	100	100		0	0	0	0	0	0	0	0	0	0	0	0	2	0	0
OP974531 SD-15/Goat	100	100	100	100	100	99.6	100	100	100	100	100		0	0	0	0	0	0	0	0	0	0	0	2	0	0
OP974532 SD-16/Goat	100	100	100	100	100	99.6	100	100	100	100	100	100		0	0	0	0	0	0	0	0	0	0	2	0	0
OP974533 SD-17/Goat	100	100	100	100	100	99.6	100	100	100	100	100	100	100		0	0	0	0	0	0	0	0	0	2	0	0
OP974534 SD-18/Goat	100	100	100	100	100	99.6	100	100	100	100	100	100	100	100		0	0	0	0	0	0	0	0	2	0	0
OP974535 SD-20/Goat	100	100	100	100	100	99.6	100	100	100	100	100	100	100	100	100		0	0	0	0	0	0	0	2	0	0
OP974536 SD-21/Goat	100	100	100	100	100	99.6	100	100	100	100	100	100	100	100	100	100		0	0	0	0	0	0	2	0	0
OP974537 SD-22/Goat	100	100	100	100	100	99.6	100	100	100	100	100	100	100	100	100	100	100		0	0	0	0	0	2	0	0
OP974538 SD-23/Goat	100	100	100	100	100	99.6	100	100	100	100	100	100	100	100	100	100	100	100		0	0	0	0	2	0	0
OP974539 SD-25/Goat	100	100	100	100	100	99.6	100	100	100	100	100	100	100	100	100	100	100	100	100		0	0	0	2	0	0
OP974540 SD-26/Goat	100	100	100	100	100	99.6	100	100	100	100	100	100	100	100	100	100	100	100	100	100		0	0	2	0	0
OP974541 SD-27/Goat	100	100	100	100	100	99.6	100	100	100	100	100	100	100	100	100	100	100	100	100	100	100		0	2	0	0
OP974542 SD-29/Goat	100	100	100	100	100	99.6	100	100	100	100	100	100	100	100	100	100	100	100	100	100	100	100		2	0	0
OP974582 GZ-3/Goat	99.8	99.8	99.8	99.8	99.8	99.4	99.8	99.8	99.8	99.8	99.8	99.8	99.8	99.8	99.8	99.8	99.8	99.8	99.8	99.8	99.8	99.8	99.8		2	2
OP974583 GZ-5/Goat	100	100	100	100	100	99.6	100	100	100	100	100	100	100	100	100	100	100	100	100	100	100	100	100	99.8		0
OP974584 GZ-11/Goat	100	100	100	100	100	99.6	100	100	100	100	100	100	100	100	100	100	100	100	100	100	100	100	100	99.8	100	

Appendix Figure 3. The comparison between each *groEL* gene sequences of *Anaplasma capra* this study and sequence from human. The upper right part represents the number of bases that differ from each sequence and the lower left part represents the nucleotide identity (%) between each sequence from others.

	HLJ-14	SD-1/Goat	SD-2/Goat	SD-3/Goat	SD-4/Goat	SD-6/Goat	SD-10/Goat	SD-11/Goat	SD-12/Goat	SD-13/Goat	SD-15/Goat	SD-16/Goat	SD-18/Goat	SD-19/Goat	SD-20/Goat	SD-21/Goat	SD-22/Goat	SD-23/Goat	SD-27/Goat	SD-28/Goat	SD-29/Goat	GZ-1/Goat	GZ-8/Goat	GZ-10/Goat	GZ-11/Goat	GZ-13/Goat	GZ-1/*Hae. Longicornis*	GZ-2/*Hae. longicornis*	GZ-6/*Hae. longicornis*
KM206273 HLJ-14		7	0	10	11	6	0	0	0	0	0	1	0	0	1	0	0	0	0	6	0	8	1	7	0	0	0	0	7
OQ148549 SD-1/Goat	99.5		7	1	1	1	7	6	7	7	7	7	7	7	7	7	7	7	7	2	7	8	1	6	6	6	7	7	2
OQ148550 SD-2/Goat	100	99.5		10	11	6	0	0	0	0	0	1	0	0	1	0	0	0	0	6	0	8	1	7	0	0	0	0	7
OQ148551 SD-3/Goat	99.3	99.9	99.3		9	4	10	10	10	10	10	9	10	10	11	10	10	10	10	4	10	8	1	7	10	10	10	10	0
OQ148552 SD-4/Goat	99.2	99.9	99.2	99.4		5	11	11	11	11	11	12	11	11	12	11	11	11	11	7	11	8	1	12	11	11	11	11	1
OQ148553 SD-6/Goat	99.6	99.9	99.6	99.7	99.7		6	6	6	6	6	7	6	6	7	6	6	6	6	2	6	8	1	7	6	6	6	6	1
OQ148554 SD-10/Goat	100	99.5	100	99.3	99.2	99.6		0	0	0	0	1	0	0	1	0	0	0	0	6	0	8	1	7	0	0	0	0	7
OQ148555 SD-11/Goat	100	99.6	100	99.3	99.2	99.6	100		0	0	0	1	0	0	1	0	0	0	0	6	0	8	1	7	0	0	0	0	7
OQ148556 SD-12/Goat	100	99.5	100	99.3	99.2	99.6	100	100		0	0	1	0	0	0	0	0	0	0	6	0	8	1	7	0	0	0	0	7
OQ148557 SD-13/Goat	100	99.5	100	99.3	99.2	99.6	100	100	100		0	1	0	0	1	0	0	0	0	6	0	8	1	7	0	0	0	0	7
OQ148558 SD-15/Goat	100	99.5	100	99.3	99.2	99.6	100	100	100	100		1	0	0	1	0	0	0	0	6	0	8	1	7	0	0	0	0	7
OQ148559 SD-16/Goat	99.9	99.5	99.9	99.4	99.2	99.5	99.9	99.9	99.9	99.9	99.9		1	1	2	1	1	1	1	5	1	8	1	6	1	1	1	1	7
OQ148560 SD-18/Goat	100	99.5	100	99.3	99.2	99.6	100	100	100	100	100	99.9		0	1	0	0	0	0	6	0	8	1	7	0	0	0	0	7
OQ148561 SD-19/Goat	100	99.5	100	99.3	99.2	99.6	100	100	100	100	100	99.9	100		0	0	0	0	0	6	0	8	1	7	0	0	0	0	7
OQ148562 SD-20/Goat	99.9	99.5	99.9	99.2	99.2	99.5	99.9	99.9	100	99.9	99.9	99.9	99.9	100		0	1	0	1	6	1	8	1	7	0	1	1	1	7
OQ148563 SD-21/Goat	100	99.5	100	99.3	99.2	99.6	100	100	100	100	100	99.9	100	100	100		0	0	0	6	0	8	1	7	0	0	0	0	7
OQ148564 SD-22/Goat	100	99.5	100	99.3	99.2	99.6	100	100	100	100	100	99.9	100	100	99.9	100		0	0	6	0	8	1	7	0	0	0	0	7
OQ148565 SD-23/Goat	100	99.5	100	99.3	99.2	99.6	100	100	100	100	100	99.9	100	100	100	100	100		0	6	0	8	1	7	0	0	0	0	7
OQ148566 SD-27/Goat	100	99.5	100	99.3	99.2	99.6	100	100	100	100	100	99.9	100	100	99.9	100	100	100		6	0	8	1	7	0	0	0	0	7
OQ148567 SD-28/Goat	99.6	99.9	99.6	99.7	99.5	99.9	99.6	99.6	99.6	99.6	99.6	99.6	99.6	99.6	99.6	99.6	99.6	99.6	99.6		6	8	1	6	6	6	6	6	2
OQ148568 SD-29/Goat	100	99.5	100	99.3	99.2	99.6	100	100	100	100	100	99.9	100	100	99.9	100	100	100	100	99.6		8	1	7	0	0	0	0	7
OQ148572 GZ-1/Goat	99.4	99.4	99.4	99.4	99.4	99.4	99.4	99.4	99.4	99.4	99.4	99.4	99.4	99.4	99.4	99.4	99.4	99.4	99.4	99.4	99.4		9	8	8	8	8	8	8
OQ148573 GZ-8/Goat	99.9	99.9	99.9	99.9	99.9	99.9	99.9	99.9	99.9	99.9	99.9	99.9	99.9	99.9	99.9	99.9	99.9	99.9	99.9	99.9	99.9	99.3		7	1	1	1	1	1
OQ148574 GZ-10/Goat	99.5	99.5	99.5	99.5	99.1	99.5	99.5	99.5	99.5	99.5	99.5	99.6	99.5	99.5	99.5	99.5	99.5	99.5	99.5	99.6	99.5	99.4	99.5		7	7	7	7	6
OQ148575 GZ-11/Goat	100	99.6	100	99.3	99.2	99.6	100	100	100	100	100	99.9	100	100	100	100	100	100	100	99.6	100	99.4	99.9	99.5		0	0	0	7
OQ148576 GZ-13/Goat	100	99.6	100	99.3	99.2	99.6	100	100	100	100	100	99.9	100	100	99.9	100	100	100	100	99.6	100	99.4	99.9	99.5	100		0	0	7
OQ148569 GZ-1/*Hae. longicornis*	100	99.5	100	99.3	99.2	99.6	100	100	100	100	100	99.9	100	100	99.9	100	100	100	100	99.6	100	99.4	99.9	99.5	100	100		0	7
OQ148570 GZ-2/*Hae. longicornis*	100	99.5	100	99.3	99.2	99.6	100	100	100	100	100	99.9	100	100	99.9	100	100	100	100	99.6	100	99.4	99.9	99.5	100	100	100		7
OQ148571 GZ-6/*Hae. longicornis*	99.5	99.9	99.5	100	99.9	99.9	99.5	99.5	99.5	99.5	99.5	99.5	99.5	99.5	99.5	99.5	99.5	99.5	99.5	99.9	99.5	99.4	99.9	99.5	99.5	99.5	99.5	99.5	

Appendix Figure 4. The comparison between each 16S rRNA gene sequences of *Anaplasma capra* this study and sequence from human. The upper right part represents the number of bases that differ from each sequence and the lower left part represents the nucleotide identity (%) between each sequence from others.

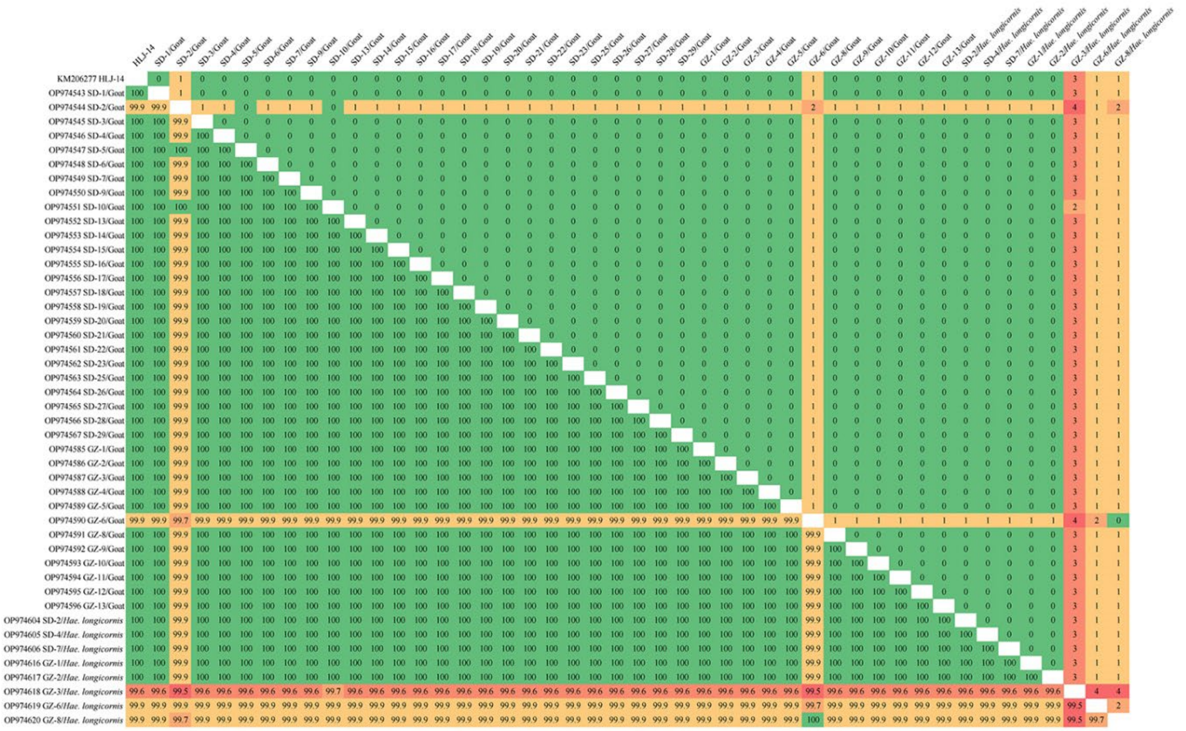

Appendix Figure 5. The comparison between each *msp4* gene sequences of *Anaplasma capra* this study and sequence from human. The upper right part represents the number of bases that differ from each sequence and the lower left part represents the nucleotide identity (%) between each sequence from others.
